# Desacetyl-α-melanocyte stimulating hormone and α-melanocyte stimulating hormone are required to regulate energy balance

**DOI:** 10.1016/j.molmet.2017.11.008

**Published:** 2017-11-24

**Authors:** Kathleen G. Mountjoy, Alexandre Caron, Kristina Hubbard, Avik Shome, Angus C. Grey, Bo Sun, Sarah Bould, Martin Middleditch, Beau Pontré, Ailsa McGregor, Paul W.R. Harris, Renata Kowalczyk, Margaret A. Brimble, Rikus Botha, Karen M.L. Tan, Sarah J. Piper, Christina Buchanan, Syann Lee, Anthony P. Coll, Joel K. Elmquist

**Affiliations:** 1 Department of Physiology, University of Auckland, Private Bag 92019, Auckland 1142, New Zealand; 2 Department of Molecular Medicine and Pathology, University of Auckland, Private Bag 92019, Auckland 1142, New Zealand; 3 Maurice Wilkins Centre for Molecular Biodiscovery, University of Auckland, Private Bag 92019, Auckland 1142, New Zealand; 4 Department of Internal Medicine, Division of Hypothalamic Research, The University of Texas Southwestern Medical Center, Dallas, TX, USA; 5 Department of Anatomy and Medical Imaging, University of Auckland, Private Bag 92019, Auckland 1142, New Zealand; 6 School of Biological Sciences, University of Auckland, Private Bag 92019, Auckland 1142, New Zealand; 7 Department of Pharmacy, University of Auckland, Private Bag 92019, Auckland 1142, New Zealand; 8 School of Chemical Sciences, University of Auckland, Private Bag 92019, Auckland 1142, New Zealand; 9 Department of Clinical Biochemistry, Cambridge Institute for Medical Research, Addenbrooke's Hospital, Cambridge CB2 2QR, United Kingdom; 10 University of Cambridge Metabolic Research Laboratories, MRC Metabolic Diseases Unit, Wellcome Trust-MRC Institute of Metabolic Science, Cambridge CB2 0QQ, United Kingdom

**Keywords:** POMC, Obesity, Desacetyl-α-MSH, α-MSH, Obese mouse model

## Abstract

**Objective:**

Regulation of energy balance depends on pro-opiomelanocortin (POMC)-derived peptides and melanocortin-4 receptor (MC4R). Alpha-melanocyte stimulating hormone (α-MSH) is the predicted natural POMC-derived peptide that regulates energy balance. Desacetyl-α-MSH, the precursor for α-MSH, is present in brain and blood. Desacetyl-α-MSH is considered to be unimportant for regulating energy balance despite being more potent (compared with α-MSH) at activating the appetite-regulating MC4R *in vitro*. Thus, the physiological role for desacetyl-α-MSH is still unclear.

**Methods:**

We created a novel mouse model to determine whether desacetyl-α-MSH plays a role in regulating energy balance. We engineered a knock in targeted QKQR mutation in the POMC protein cleavage site that blocks the production of both desacetyl-α-MSH and α-MSH from adrenocorticotropin (ACTH_1-39_).

**Results:**

The mutant ACTH_1-39_ (ACTH^QKQR^) functions similar to native ACTH_1-39_ (ACTH^KKRR^) at the melanocortin 2 receptor (MC2R) *in vivo* and MC4R *in vitro*. Male and female homozygous mutant ACTH_1-39_ (*Pomc*^tm1/tm1^) mice develop the characteristic melanocortin obesity phenotype. Replacement of either desacetyl-α-MSH or α-MSH over 14 days into *Pomc*^tm1/tm1^ mouse brain significantly reverses excess body weight and fat mass gained compared to wild type (WT) (*Pomc*^wt/wt^) mice. Here, we identify both desacetyl-α-MSH and α-MSH peptides as regulators of energy balance and highlight a previously unappreciated physiological role for desacetyl-α-MSH.

**Conclusions:**

Based on these data we propose that there is potential to exploit the naturally occurring POMC-derived peptides to treat obesity but this relies on first understanding the specific function(s) for desacetyl-α-MSH and α-MSH.

## Highlights

• KKRR → QKQR mutation in the cleavage site of POMC prevents the production of desacetyl-α-MSH and α-MSH in mice.
• Male and female mutant mice develop characteristic melanocortin obesity.
• Central administration of α-MSH is more potent at reducing body weight in female mutant mice.
• Central administration of desacetyl-α-MSH and α-MSH are similarly potent at reducing body weight in male mutant mice.

## 1 Introduction

The melanocortin system plays a significant role in the regulation of energy balance (see reviews [1], [2], [3]). However, little is known about which specific endogenous pro-opiomelanocortin (POMC)-derived peptides are responsible for regulation of appetite, metabolism, and body weight. The POMC protein is inherently complex and is differentially cleaved into multiple peptides in a coordinated and tissue-specific manner [4]. POMC is a prohormone, and its processing involves proteolytic cleavages at specific pairs of basic amino acids performed by enzymes, prohormone converting enzyme 1 (PC1), prohormone converting enzyme 2 (PC2), and carboxypeptidase E (CPE) (reviewed in Ref. [5]). In brain and pituitary pars distalis and pituitary pars intermedia, POMC is cleaved by PC1 to produce multiple peptides including ACTH_1-39_ and β-lipotrophin (β-LPH). PC2 is selectively expressed in brain and pituitary pars intermedia, and it cuts ACTH_1-39_ further at tandem dibasic residues, KKRR, to produce ACTH_1-17_ and corticotropin-like intermediate lobe peptide (CLIP). CPE subsequently removes basic amino acids at the C-terminus of ACTH_1-17_ to produce ACTH_1-13_. Post-translational processing of ACTH_1-13_ produces desacetyl-α-MSH, α-MSH (monoacetylated) and diacetyl-α MSH. PC2 also cuts β-LPH to generate γ-LPH and β-endorphin.

One POMC-derivative, β-endorphin, stimulates food intake [6], [7], [8] while four POMC-derived peptides, ACTH_1-39_, α-MSH, β-MSH, and γ2-MSH, reduce food intake [6], [9], [10]. A sixth peptide, desacetyl-α-MSH, also reduces food intake but in pharmacological studies requires a 25-times higher dose than α-MSH [9]. For this reason, desacetyl-α-MSH has been considered to be unimportant for the regulation of energy balance [5], [11], [12]. However, there is a higher abundance of desacetyl-α-MSH compared with α-MSH in rat hypothalamus [13], [14]. In addition, desacetyl-α-MSH (compared with α-MSH) is more potent at activating the appetite-regulating MC4R *in vitro* [1]. Thus, the physiological role of desacetyl-α-MSH still remains unclear.

The melanocortin peptides differentially activate five melanocortin receptor (MCR) subtypes, each having unique tissue distributions and functions. MC3R and MC4R are highly expressed in the central nervous system and play key roles in regulating energy balance [15], [16], [17]. Multiple POMC-derived peptides activate MC3R and MC4R *in vitro* [18], [19], [20]. However, it is unknown whether these peptides have distinct or redundant roles *in vivo* [2]. Since studies have indicated that only pharmacologic concentrations of desacetyl-α-MSH (compared to α-MSH) inhibit food intake [9], [21], α-MSH is predicted to be the endogenous melanocortin peptide hormone that regulates energy balance. In addition, β-MSH is not present in rodents [22]. Here, we determined the direct contribution of desacetyl-α-MSH and α-MSH in regulating energy balance.

## 2 Materials and methods

### 2.1 Generation and maintenance of *Pomc*^*tm1*^ targeted mutation mouse model

The objective of this study is to develop a mouse model with a targeted *Pomc* mutation that prevents production of desacetyl-α-MSH and α-MSH and then use this model to determine whether desacetyl-α-MSH plays a role in energy balance. Ozgene Pty Ltd. (Bentley DC, WA, Australia) generated the *Pomc*^*tm1Kgm*^^11^ knock in mouse strain, the first targeted mutation (tm1) in the mouse *Pomc* gene that prevents ACTH_1-39_ cleavage into ACTH_1-17_ and CLIP. We first validated that mutant ACTH^QKQR^ (found in *Pomc*^tm1/tm1^ mice) functions similar to wild type (WT) ACTH^KKRR^ (found in *Pomc*^wt/wt^ mice) both *in vitro* and *in vivo* (see Supplementary Data). A targeting vector was created containing mouse *Pomc* exon 3 KKRR proteolytic cleavage site mutated to QKQR with *PGK-Neo* selection cassette inserted downstream of WT exon 3. *Lox P* sites were inserted flanking WT exon 3 and the *PGK-Neo* selection cassette. The targeting vector was constructed from three fragments, the 5′ homology arm, the 3′ homology arm and the *lox P* arm, which were all generated by PCR. Cre-recombinase deletes the PGK-Neo cassette and WT exon 3 allowing the mutant QKQR exon 3 to be expressed. Following electroporation of the targeting construct into C57BL/6J Bruce4 embryonic stem (ES) cells, cells were selected for neomycin resistance. Southern blotting and PCR were used to confirm targeted ES cells. Euploid, targeted ES cells were then microinjected into *Balb/cJ* blastocysts and re-implanted into pseudo-pregnant dams. Resultant chimeras were bred to *C57BL/6J* breeders to establish transmission. Black progeny that were heterozygous for the gene-targeted allele were then bred to Cre recombinase “delete” mice on *C57BL/6J* background (Ozgene Pty Ltd.) to allow excision of the WT exon 3 and Neo selection cassette. Cre was then removed by breeding to *C57BL/6J* WT mice. Resulting mice were transferred to the Vernon Jensen Animal Unit at the University of Auckland (UOA) where the colony is maintained with heterozygous breeding pairs. Mice were transferred from the University of Auckland to University of Texas Southwestern Medical Center (UTSW) where the colony is maintained with triplicate heterozygous mouse breeding.

Routine genotyping was performed by a PCR based strategy utilizing primers that anneal to *Pomc* exon 3 (forward 5′TGCATCCGGGCTTGCAAACTCGA3′ and reverse 5′GGGGCAAGGAGGTTGAGAAAT3′) yielding an 820 bp fragment. HaeII restriction enzyme was used to cleave the 802 bp fragment to yield 514 bp, 234 bp and 54 bp fragments. The QKQR mutation destroyed one of the HaeII sites and therefore HaeII cleaved the homozygous KI to yield 568 bp and 234 bp fragments.

### 2.2 Ethics and animal husbandry

All experimental procedures involving mice at the Vernon Jensen Animal Facility, UOA, were approved by the Auckland University Animal Ethics Committee and conformed to The Animal Welfare Act 1999. Animals were housed up to 6 per cage on wood-chip bedding and maintained at room ambient 20 °C with a 12-h dark–light cycle (lights on at 07:00 h in a pathogen-free barrier facility). The mice were fed regular chow (Teklad Global 18% protein rodent diet 2018 [Harlan Laboratories, Inc., Madison, WI, USA]). All experimental procedures for the metabolic cages were performed at UTSW and were approved by the IACUC committee at UTSW. The *Pomc*^tm1Kgm^ mouse breeding colony was established at UTSW to produce mice for testing in metabolic cages. At UTSW, mice were bred and housed in a barrier facility at room ambient 22–24 °C on a 12 h light/12 h dark cycle and were provided standard chow (2016; Harlan Teklad) as well as water ad libitum. All experimental procedures involving mice at University of Cambridge were carried out in accordance with the guidelines of the United Kingdom Home Office. Animals were kept under controlled temperature (22 °C) and 12 h light, 12 h dark schedule (lights on 7:00–19.00).

### 2.3 Growth and development

Groups comprising *Pomc*^wt/wt^, *Pomc*^wt/tm1^ and *Pomc*^tm1/tm1^ mice of each sex were weighed biweekly from weaning until 19–20 weeks of age. Significant differences were determined using two-way repeated-measures ANOVA and Bonferroni post-hoc test. Examination of both sexes allowed for assessment of sexually dimorphic phenotypes. At 27–30 weeks, the mice were fasted overnight before being euthanized with isoflurane, blood collected by cardiac puncture and nose-anus and anus-tail tip measurements recorded. Significant differences were determined using one-way ANOVA and Tukey's post-hoc test.

### 2.4 Body composition

Body composition was analyzed by magnetic resonance imaging (MRI) at the UOA and nuclear magnetic resonance (NMR) at UTSW. MRI was used to assess body composition of *Pomc*^wt/wt^, *Pomc*^wt/tm1^ and *Pomc*^tm1/tm1^ mice and to compare body composition of male *Pomc*^tm1/tm1^ mice following melanocortin peptide treatment. NMR (minispec, Bruker) was used to compare body composition prior to metabolic cage experiments. MRI was performed using a 4.7T horizontal bore magnet interfaced with a UnityInova spectrometer (Agilent Technologies, Santa Clara, CA, USA). The anesthetized animals were placed in a 72 mm ID circularly-polarized radio-frequency coil for imaging (m2m Imaging, Cleveland, OH, USA). Localizer images were used to determine the appropriate position and number of slices to ensure that all of the animal's tissue was included in the body composition assessment. The scans to determine the body composition of the animals used the three-point Dixon technique [23] on a set of contiguous, 1 mm thick slices with a field-of-view of 110 × 55 mm and the imaging matrix set to 256 × 128. The repetition time (TR) was 1000 ms and the echo times were specified so that one in-phase image (0°), and two out-of-phase images (−180°, 180°) were acquired. All image processing to extract the fat and lean-tissue images from the MRI data and to determine the body composition was performed with MATLAB (Mathworks Inc., Natick, MA, USA) using previously described techniques [23]. Significant differences were determined using one-way ANOVA and Tukey's post-hoc test.

### 2.5 Metabolic cages

Metabolic measurements were obtained for male and female *Pomc*^wt/wt^ and *Pomc*^tm1/tm1^ mice aged ∼4–6 weeks fed a regular chow diet or a regular chow diet and switched to a high-fat diet for the duration of the time they were housed in metabolic cages. Before each experiment, body composition of ad libitum fed mice was assessed using NMR spectrometer, and the mice were acclimatized to individual caging for 3–4 days. Mice were then transferred to metabolic chambers for an additional 4-day acclimatization period with food provided ad libitum. Following acclimatization, energy expenditure (O_2_ consumption) was measured by indirect calorimetry and simultaneous locomotor activity was assessed by infrared light-beam frame surrounding the cage using TSE Labmaster monitoring system (TSE Systems GmbH, Bad Homburg, Germany). Average oxygen consumption was calculated for both light and dark periods and expressed per total or lean body mass. For locomotor activity analysis, beam breaks in X- and Y-axis (ambulatory activity) were measured and summed over dark and light periods. Significant differences were determined using two-way repeated measures ANOVA and Bonferroni post-hoc analysis or unpaired two-tail Student's *t* test.

### 2.6 Central melanocortin peptide treatment

We administered melanocortin peptides to mice continuously using osmotic mini pumps but first we determined using MALDI–TOF MS that α-MSH and desacetyl-α-MSH dissolved in PBS and stored at 37 °C were stable over 14 days. Aliquots of α-MSH and desacetyl-α-MSH dissolved in PBS that were prepared for treatment studies were incubated in Lo-bind Eppendorf tubes at 37 °C. At 7, 10, and 14 days, aliquots were snap frozen at −80 °C. After thawing, the aliquots were centrifuged at 13,000g for 2 min at 4 °C. Spots (1 μL) of each supernatant were then spiked on a MALDI–TOF plate and dried for ≥30 min in a vacuum desiccator. Matrix (αCyano-4-hydroxycinnamic acid in 50% acetonitrile in sterile water with 0.1% TFA) was applied manually over peptides and allowed to thoroughly dry before the plate was read in a Voyager DE-Pro Mass Spectrometer (Applied Biosystems). After dissolving in PBS, melanocortin peptides were primed overnight at 37 °C in osmotic mini pumps before being administered intracerebroventricular (i.c.v.) continuously over 14 days by osmotic mini pump infusions. Group-housed mice (*n* = 3–6 mice per cage) underwent stereotaxic surgery under isoflurane anesthesia to implant a cannula into the lateral cerebral ventricle with the following coordinates: anterior posterior 0.1 mm, medial lateral 0.9 mm with one spacer dorsal ventral. An Alzet^®^ mini osmotic pump (Model 1002, Bio-Scientific Pty Ltd., NSW, Australia) filled either with saline vehicle (USP-IV-IM, Demo Pharmaceutical Industry, Greece) or melanocortin peptide (delivering 0.05 μg, 0.5 μg or 5 μg of peptide/25 g mouse body weight/day) was implanted subcutaneously and attached to the cannula using a catheter (Alzet Brain Infusion Kit 3, Bio-Scientific Pty Ltd.). Mice were allowed to recover from surgery for ∼2–4 h before being returned to their group-housed cages. Individual body weights and food and water intake for each cage were monitored daily over 14 days. All mice were monitored daily for signs of ill health (not eating, starry-fur, not moving). Significant differences were determined using two-way repeated measures ANOVA and Dunnett's post-hoc analysis.

### 2.7 Statistical analysis

GraphPad Prism 7 software (GraphPad Software Inc., San Diego, CA) was used to perform all statistical analyses. Comparisons between groups were made by two-way or one-way repeated or non-repeated measures ANOVA with Tukey or Bonferroni post-hoc analysis, or by 2-tailed Student ‘*t*’ test as indicated. Changes in body weight over time comparisons were made using repeated two-way ANOVA. *P* < 0.05 was considered statistically significant. Data are presented as mean ± SEM.

## 3 Results

### 3.1 A *Pomc* gene targeted mutation (*Pomc*^*tm1*^) results in biologically active QKQR mutant ACTH_1-39_ hormone

**Figure 1:**
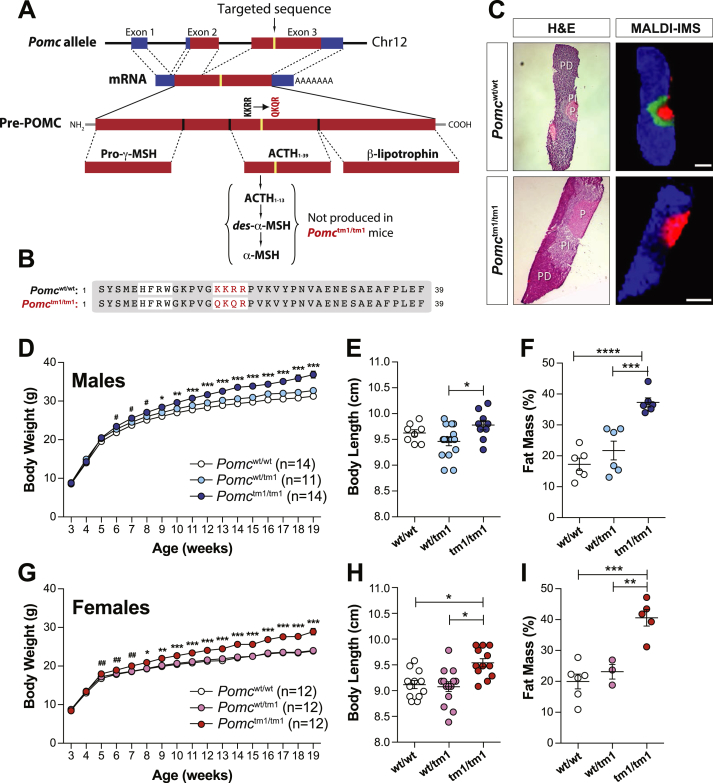
**Generation of *Pomc*^tm1/tm1^ mice that develop the characteristic melanocortin obese phenotype. A**, Schematic of targeted *Pomc* allele for knock-in of QKQR mutation into *Pomc* exon 3 with resulting impact on pre-POMC processing and ACTH_1-13_ production. **B**, Amino acid sequence alignments for native and mutant ACTH_1-39_ molecule. **C**, MALDI imaging MS shows ACTH_1-13_ is successfully deleted from *Pomc*^tm1/tm1^ mouse pituitary. Mass-to-charge (*m*/*z*) signals that delineate the pars distalis (PD, *m*/*z* 835 in *blue* represents phospholipid) and posterior lobe (P, *m*/*z* 1086 in *red* represents vasopressin) are shown. In addition, diacetyl-α-MSH (*m*/*z* 1706 in *green*) is detected in the pars intermedia (PI) of *Pomc*^wt/wt^ but not *Pomc*^tm1/tm1^ tissue. Scale bars = 500 μM. **D and G**, Body weights of mice fed a regular-chow diet from weaning. Significant difference determined using two-way repeated-measures ANOVA and Bonferroni post-hoc test between *Pomc*^wt/wt^ and *Pomc*^tm1/tm1^. *, *p* < 0.05; **, *p* < 0.01; ***, *p* < 0.001 or using paired Student ‘*t*’ test between *Pomc*^wt/wt^ and *Pomc*^tm1/tm1^; male ^#^, *p* < 0.05; female ^##^, *p* < 0.01. **E and H**, Body length measured at 27–30 weeks for mice fed a regular-chow diet from weaning. Data are shown as mean ± SEM. Significant differences determined using one-way ANOVA and Tukey's post-hoc test. *, *p* < 0.05; **, *p* < 0.01. **F and I**, Percent body fat calculated from 6 MRI Dixon images/mouse. Data are shown as mean ± SEM for mice aged 26–29 weeks and fed a regular-chow diet. Significant differences determined using one-way ANOVA and Tukey's post-hoc test. ***, *p* < 0.001; ****, *p* < 0.0001.

Deletion in the *Pomc* gene results in obesity in both mice [24], [25], [26] and humans [27]. However, the *Pomc* null mouse is not suitable for determining specific POMC-derived peptide functions since it lacks all POMC-derived peptides and does not develop functional adrenal glands [24], [26], [28]. Thus, we developed a unique mouse model (*Pomc*^*tm1Kgm*^) with a targeted QKQR mutation in the POMC protein cleavage site that is required to produce desacetyl-α-MSH and α-MSH from ACTH_1-39_ (Figure 1A).

We performed a series of biochemical and physiological studies to validate biological activity for QKQR mutant ACTH_1-39_ (ACTH^QKQR^, see amino acid alignment, Figure 1B). ACTH^QKQR^ stimulates corticosterone production similar to native ACTH_1-39_ (ACTH^KKRR^) in dexamethasone-suppressed *Pomc*^wt/wt^ male mice (Supplementary Figure 1A). The ACTH^QKQR^, like native ACTH^KKRR^, is biologically active at the MC4R *in vitro* (Supplementary Figure 1B). *Pomc*^tm1/tm1^ mice develop functional adrenal glands and produce corticosterone levels similar to *Pomc*^wt/wt^ mice (Supplementary Figure 1C). These results confirm that ACTH^QKQR^ is produced and functional in *Pomc*^tm1/tm1^ mice.

### 3.2 ACTH^QKQR^ protein is not cleaved to produce desacetyl-α-MSH and α-MSH

We chose pituitary to validate that the QKQR mutation blocks ACTH_1-39_ cleavage *in vivo* because POMC is abundantly expressed in pituitary pars distalis and pars intermedia while lesser amounts of POMC are expressed in the arcuate nucleus of the hypothalamus. The pituitary pars intermedia is a good surrogate for the arcuate nucleus since they both express PC2, the enzyme required for cleaving ACTH_1-39_ to ACTH_1-17_. The pars distalis and posterior lobe of the pituitary are helpful controls since the pars distalis expresses POMC but no PC2 while the posterior lobe of the pituitary does not express either POMC or PC2.

To validate that ACTH^QKQR^ protein is not cleaved, we used Matrix Assisted Laser Desorption/Ionization (MALDI)–Time-of-Flight (TOF) Mass Spectrometry (MS) of pituitary sections and lysates (see Supplementary Methods). MALDI–TOF MS imaging of pituitary sections confirms that diacetyl-α-MSH is present in *Pomc*^wt/wt^ but not in *Pomc*^tm1/tm1^ pars intermedia, while phospholipid (marker for pars distalis) [29] and vasopressin (marker for posterior pituitary lobe) [29] are present in the pars distalis and posterior lobe respectively, of both *Pomc*^wt/wt^ and *Pomc*^tm1/tm1^ mice (Figure 1C). In addition, a signal predicted to be Arg-CLIP (1–22; cleaved from the C-terminus of ACTH_1-39_) is only detectable in *Pomc*^wt/wt^ whole pituitary lysate (Supplementary Figure 2A), while vasopressin, J peptide and a signal predicted to be β-LPH appear in both *Pomc*^wt/wt^ and *Pomc*^tm1/tm1^ whole pituitary lysate (Supplementary Figure 2A and B). ACTH_1-39_ and β-LPH are the predominant POMC-derived peptides produced in pars distalis and diacetyl-α-MSH, α-MSH and β-LPH are the predominant POMC-derived peptides produced in pars intermedia. β-endorphin was not detected here, but, under conditions of stress, β-LPH in pars intermedia is cleaved by PC2 to produce β-endorphin [30]. Thus, in the *Pomc*^tm1/tm1^ mouse only the ACTH^QKQR^ is not cleaved *in vivo* to produce ACTH_1-13_ and Arg-CLIP, while all other melanocortin peptides are produced through *in vivo* cleavage.

### 3.3 N-terminal acetylation of ACTH^QKQR^ protein in whole pituitary lysate

Surprisingly, MALDI–TOF MS showed a clear signal at *m*/*z* 4638 that appears only in *Pomc*^tm1/tm1^ and not in *Pomc*^wt/wt^ whole pituitary lysate (Supplementary Figure 2B). We identified this peptide as N-terminal acetylated ACTH^QKQR^ using immunoprecipitation and LC–MS/MS. We determined that acetylation of ACTH^QKQR^ does not change ACTH^QKQR^ functional coupling at the mouse MC4R *in vitro* and it abolishes ACTH^QKQR^ functional coupling of the mouse MC2R (Supplementary Figure 3A and B). Therefore, acetyl-ACTH^QKQR^ produced in pituitary, presumably in pars intermedia where desacetyl-α-MSH is normally acetylated, is not expected to affect the phenotype of *Pomc*^tm1/tm1^ mice.

### 3.4 Male and female *Pomc*^tm1/tm1^ mice develop characteristic melanocortin obesity

Despite expressing non-acetylated and acetylated ACTH^QKQR^, both of which functionally couple to the mouse MC4R *in vitro*, male and female *Pomc*^tm1/tm1^ mouse body weights are significantly increased compared to *Pomc*^wt/wt^ and *Pomc*^wt/tm1^ mice starting at 4–6 weeks of age (Figure 1D,G), due to increased lean and fat mass. Female and male *Pomc*^tm1/tm1^ body lengths are ∼5% and ∼3% longer, respectively, compared to *Pomc*^wt/wt^ or *Pomc*^wt/tm1^ mice (Figure 1E,H). Quantitative magnetic resonance imaging (MRI) analysis of whole-body tissue composition at 26–29 weeks shows significant increases in fat mass in *Pomc*^tm1/tm1^ male and *Pomc*^tm1/tm1^ female mice compared with *Pomc*^wt/wt^ mice (Figure 1F,I). These results indicate that the absence of desacetyl-α-MSH and α-MSH is sufficient to induce the characteristic melanocortin obesity phenotype, attributed to increased fat and lean mass as well as increased body length.

### 3.5 *Pomc*^tm1/tm1^ mouse hyperphagia is exacerbated when mice are fed high-fat diet

**Figure 2:**
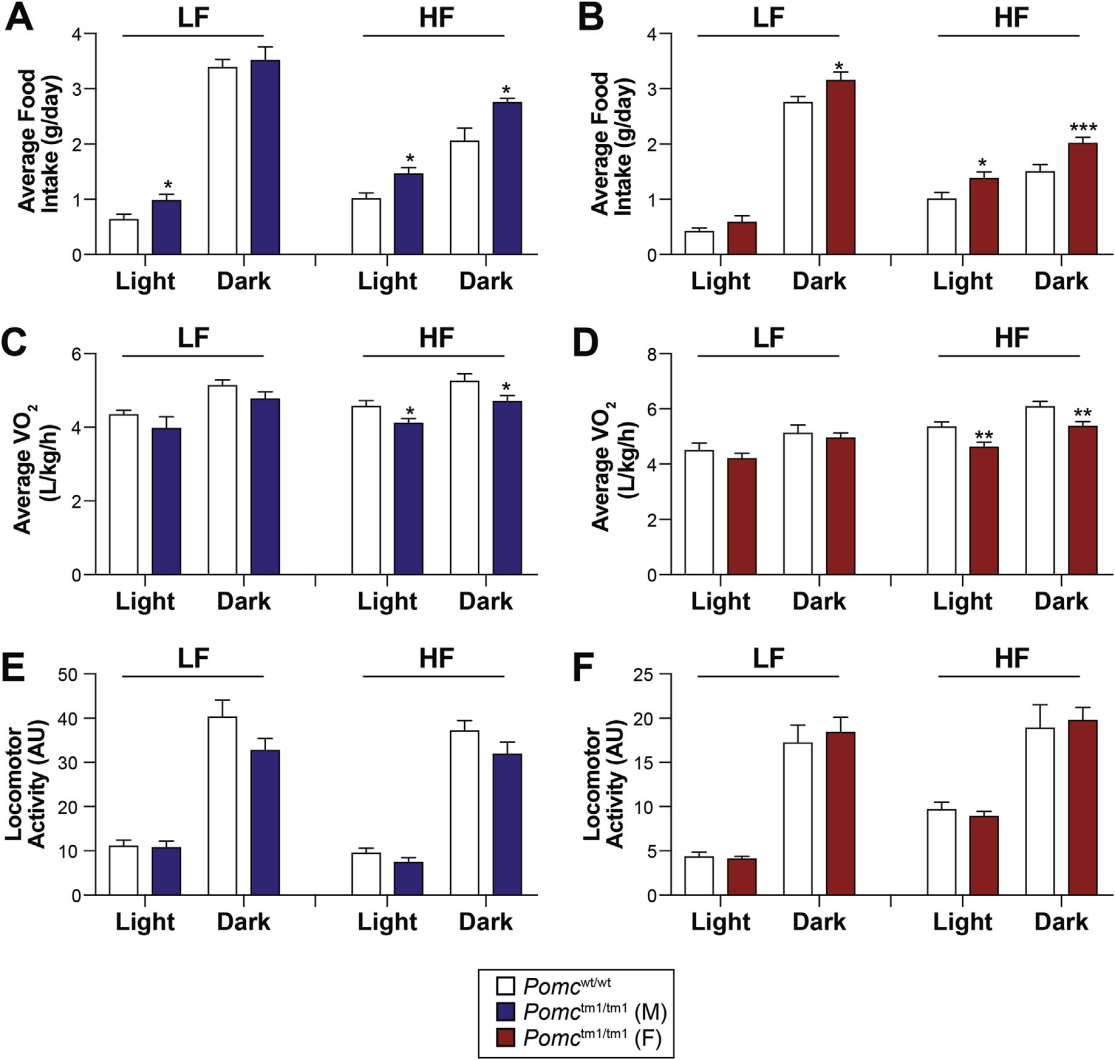
**Food intake and energy expenditure for male and female *Pomc*^wt/wt^ and *Pomc*^tm1/tm1^ mice. A and B**, Food intake was automatically measured in metabolic cages for mice at 4 weeks of age and fed regular chow for 4 days and then switched to high-fat diet for 4 days (*n* = 5–6 mice/group). Mice were acclimatized to the metabolic cages for 5 days prior to experiments. Data are shown as average food intake ± SEM per light cycle over 4 consecutive days for males and females. Significant differences determined using either two-way repeated measures ANOVA and Bonferroni post-hoc analysis or unpaired two-tail Student's *t* test. *, *p* < 0.05; ***, *p* < 0.001. **C and D**, Oxygen consumption (VO_2_) measured in metabolic cages for the same mice shown in A and B. Data shown as average VO_2_ per light cycle ± SEM over 4 consecutive days for males and females. Significant differences determined using either two-way repeated measures ANOVA and Bonferroni post-hoc analysis or unpaired two-tail Student's *t* test. *, *p* < 0.05; **, *p* < 0.01. **E and F**, Locomotor activity measured in metabolic cages for same mice as shown in A and B. Data are shown as total activity per light cycle ± SEM over 4 consecutive days for males and females. No significant differences were determined using either two-way repeated measures ANOVA and Bonferroni post-hoc analysis or unpaired two-tail Student's *t* test.

We next sought to determine what parameters of energy balance are altered and cause obesity in early age. Mice (4 weeks of age) were individually housed in metabolic cages to investigate how the absence of desacetyl-α-MSH and α-MSH affects feeding behavior and energy expenditure, before differences in body weight might confound interpretation. While all *Pomc*^tm1/tm1^ mice exhibit hyperphagia, we observed that male *Pomc*^tm1/tm1^ mice fed a low-fat diet (LFD) have increased food intake during the light phase, while females are hyperphagic during the dark phase (Figure 2A,B). This suggests that male *Pomc*^tm1/tm1^ mice have an altered feeding pattern, with abnormal food intake during the light-cycle. A deficiency in POMC or MC4R associates with hyperphagia that is exaggerated by dark-cycle food consumption (reviewed in Refs. [31], [32]) and is sensitive to dietary fat content [33], [34]. Here, we show high-fat diet (HFD) exacerbates hyperphagia in male and female *Pomc*^tm1/tm1^ mice throughout the day (Figure 2A,B), suggesting that the absence of desacetyl-α-MSH and α-MSH promotes food intake and potentially increases the palatability of HFD.

### 3.6 High-fat diet reduced energy expenditure for male and female *Pomc*^tm1/tm1^ mice

Manipulations of the melanocortin system were previously shown to impair energy expenditure, thus contributing to the obesity phenotype [34], [35]. Here, we observed that neither oxygen consumption nor locomotor activity was significantly altered in mice fed a LFD (Figure 2C–F). Interestingly, male and female *Pomc*^tm1/tm1^ mice fed HFD exhibit significantly reduced oxygen consumption compared to *Pomc*^wt/wt^ mice (Figure 2C,D), without changes in locomotor activity (Figure 2E,F). These data suggest that *Pomc*^tm1/tm1^ mice have reduced energy expenditure when exposed to a HFD regimen.

### 3.7 Central administration of either desacetyl-α-MSH or α-MSH reverses *Pomc*^tm1/tm1^ mouse obesity

**Figure 3:**
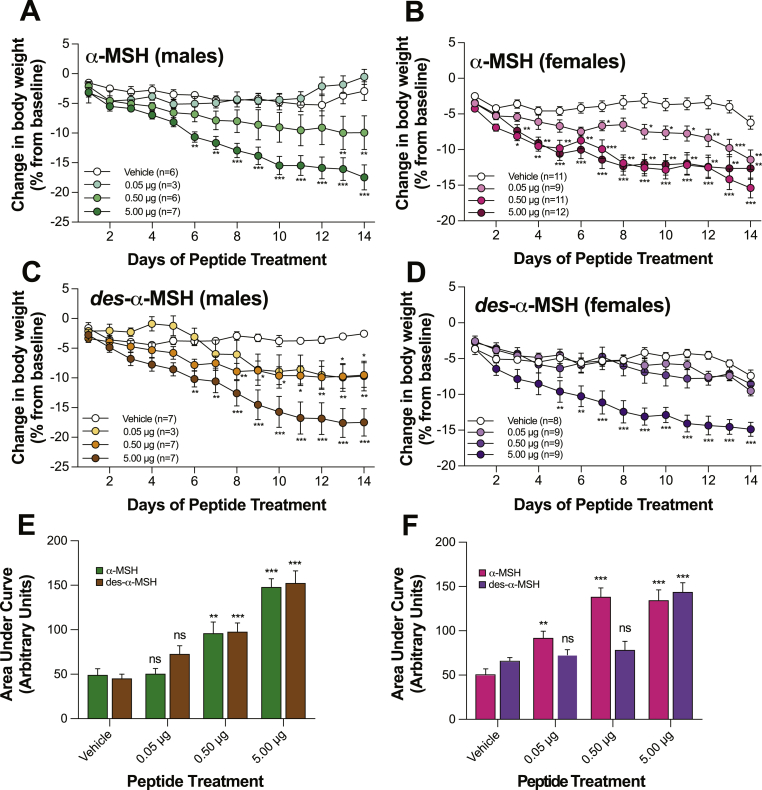
**Central α-MSH or desacetyl-α-MSH treatments reduce male and female *Pomc*^tm1/tm1^ mouse body weight. A, B, C, D, E, and F**, Administration (i.c.v.) of α-MSH or desacetyl-α-MSH compared to vehicle treatment reduced *Pomc*^tm1/tm1^ mouse body weight. At the start of treatment, male mice were aged 23–31 weeks, and female mice were aged 29–31 weeks. Vehicle or peptide dose (μg/25 g mouse body weight on day1/day) was continuously administered over 14 days. Combined data are shown as mean ± SEM for two independent experiments. **A–D**: Significant differences determined using two-way repeated measures ANOVA and Dunnett's post-hoc analysis. **E, F**: Significant differences compared to vehicle treatment determined using two-way ANOVA and Dunnett's post-hoc analysis. *, *p* < 0.05; **, *p* < 0.01; ***, *p* < 0.001.

**Figure 4:**
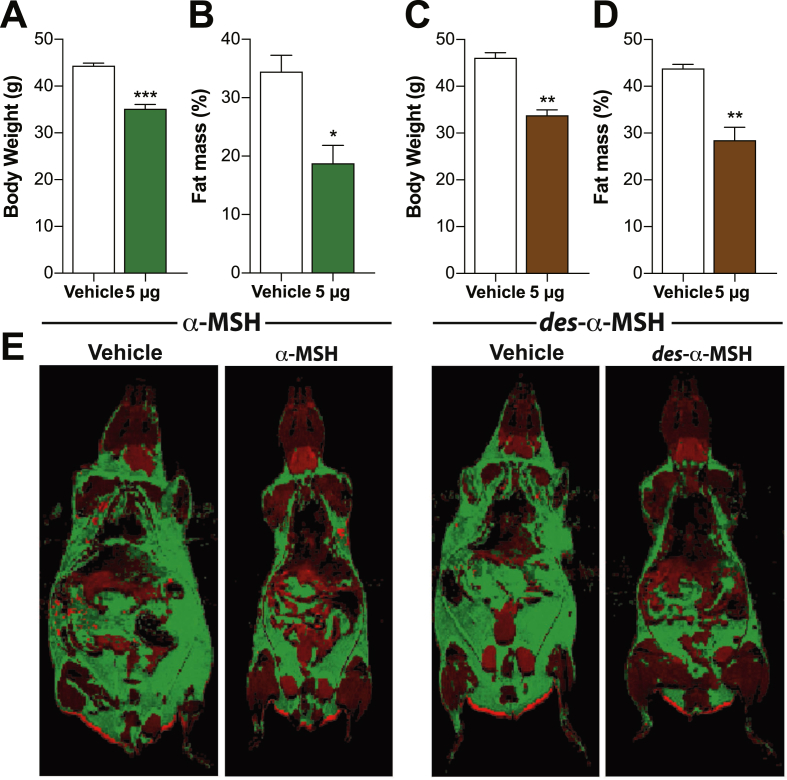
**Central α-MSH or desacetyl-α-MSH treatment reduces male *Pomc*^tm1/tm1^ mouse fat mass. A and C**, Mean body weight ± SEM for male *Pomc*^tm1/tm1^ mice (*n* = 3 group) after 14 days i.c.v. administration of vehicle, α-MSH, or desacetyl-α-MSH. **B and D**, Percent body fat ± SEM determined by MRI for male *Pomc*^tm1/tm1^ mice shown in A and C after 14 days i.c.v administration of vehicle, α-MSH, or desacetyl-α-MSH. Significant differences between vehicle and peptide treatment determined using unpaired, two-tailed Student's *t* test. *, *p* < 0.05; **, *p* < 0.01; ***, *p* < 0.001. **E**, Representative MRI images for mice presented in A–D. Fat and lean tissues represented as green and red, respectively.

To determine whether replacement of each peptide alone can reverse the characteristic melanocortin obesity, we continuously administered incremental doses (0.03–3.00 nmol/25 g body weight/day) of α-MSH or desacetyl-α-MSH into adult *Pomc*^tm1/tm1^ mouse brains over 14 days. First, we determined that α-MSH and desacetyl-α-MSH are stable under these treatment conditions (Supplementary Figure S4). We show that either α-MSH or desacetyl-α-MSH can significantly reduce body weight in *Pomc*^tm1/tm1^ mice compared with vehicle-treated age- and sex-matched control *Pomc*^tm1/tm1^ mice. Treatment with 5 μg α-MSH or 5 μg desacetyl-α-MSH similarly reduced male or female body weight (Figure 3). However, α-MSH is more potent than desacetyl-α-MSH at reducing female body weight since body weight was significantly reduced following either 0.05 μg or 0.50 μg α-MSH but not by corresponding desacetyl-α-MSH doses (Figure 3B,D, F). In contrast with females, α-MSH is not more potent than desacetyl-α-MSH at decreasing male *Pomc*^tm1/tm1^ mouse body weight; furthermore, there is a trend for desacetyl-α-MSH to be more potent than α-MSH (0.05 μg and 0.50 μg doses) at reducing male body weight (Figure 3A,C, E). The decreased body weight is predominantly due to fat mass loss: body weight and percent body fat measured using MRI in male *Pomc*^tm1/tm1^ mice treated with either α-MSH or desacetyl-α-MSH are significantly reduced compared with vehicle-treated age-matched male *Pomc*^tm1/tm1^ mice (Figure 4). The mice exhibited no signs of ill health over the 14 days of treatment; therefore, these hormones do not appear to have any non-specific toxic effects.

## 4 Discussion

The long-held myth that desacetyl-α-MSH is biologically unimportant for body weight regulation can now be put to rest. Our novel *Pomc*^tm1/tm1^ mouse identifies both desacetyl-α-MSH and α-MSH as necessary for regulating mouse energy balance. We show that preventing the production of ACTH_1-13_ from ACTH_1-39_ results in a characteristic melanocortin obesity phenotype. Furthermore, pharmacological administration of desacetyl-α-MSH or α-MSH is sufficient to reverse this phenotype.

Previously, central α-MSH administration has been shown to decrease rodent food intake and body weight [10], [36], [37], but we are the first to show potent effects for desacetyl-α-MSH decreasing mouse body weight. We show this because, in our study, desacetyl-α-MSH is administered to a mouse that does not make any endogenous desacetyl-α-MSH or α-MSH. This leads to the question as to why central administration of desacetyl-α-MSH in *Pomc*^wt/wt^ rodents does not decrease food intake similar to α-MSH [9]. We hypothesize that endogenous desacetyl-α-MSH and α-MSH prevent exogenously administered desacetyl-α-MSH from reducing food intake and body weight in *Pomc*^wt/wt^ rodents. We propose that the balance between endogenous desacetyl-α-MSH and α-MSH levels dictates the regulation of mammalian energy homeostasis and furthermore we propose the balance of these peptides could be sexually dimorphic. Here we show sensitivity to desacetyl-α-MSH and α-MSH induced weight loss differs between the sexes; male mice exhibit similar sensitivity to desacetyl-α-MSH and α-MSH while female mice are more sensitive to α-MSH compared with desacetyl-α-MSH. This adds to a list of sexually dimorphic differences reported for POMC-derived peptide regulation of energy homeostasis [38], [39], [40], [41], [42].

Leptin has been shown to stimulate N-terminal acetylation of desacetyl-α-MSH to generate α-MSH in the rodent hypothalamus [12]. α-MSH is believed to be the biologically active melanocortin hormone mediating leptin inhibition of food intake because desacetyl-α-MSH, compared with α-MSH, was shown to rapidly degrade in the hypothalamus [12]. However, our study shows that desacetyl-α-MSH and α-MSH are similarly effective at reducing *Pomc*^tm1/tm1^ mouse body weight when continuously infused at physiological levels into the lateral ventricle. Guo et al. measured ∼0.15 pmol α-MSH and ∼0.58 pmol desacetyl-α-MSH in C57BL/6J mouse hypothalamus [12]. The lowest effective dose of either hormone that we infused i.c.v. into a 35 g mouse is 0.029 pmol/min; therefore, if desacetyl-α-MSH is rapidly degraded *in vivo*, it must trigger a rapid response prior to degradation. Importantly, we determined that both α-MSH and desacetyl-α-MSH are stable when stored in PBS at 37 °C for 14 days, which are the *in vivo* conditions for the osmotic mini pumps. Therefore, in our study the osmotic mini pumps should always be pumping intact hormones.

Our data also suggest for the first time that ACTH_1-39_ is not sufficient to regulate mouse body weight despite ACTH_1-39_ having full agonist activity at the MC4R (Supplementary Figure 3A) and the ability of exogenous ACTH_1-24_ administered to rodent brain to cause decreased food intake [43]. However, it is unclear whether endogenous ACTH_1-39_ is produced in the brain and if it is, it may not be expressed when and where MC4R are expressed. The major end-products of POMC processing detected in brain hypothalamus are desacetyl-α-MSH and β-endorphin [44], [45] while α-MSH and acetylated β-endorphin expression predominate in the brainstem [44]. Hence, *Pomc*^tm1/tm1^ mouse brain is expected to express acetyl-ACTH^QKQR^ in brainstem and yet this is not sufficient to regulate *Pomc*^tm1/tm1^ mouse body weight. The acetylation reaction required for producing α-MSH is documented to occur at desacetyl-α-MSH N-terminus [4], [44], [45]. However, here we show that N-terminal acetylation occurs on ACTH_1-39_ when cleavage of ACTH_1-39_ to ACTH_1-17_ is prevented. Therefore, in the *Pomc*^tm1/tm1^ mouse, all cells and tissues that should normally express α-MSH are expected to express acetyl-ACTH^QKQR^.

A disadvantage for our novel model is that the QKQR ACTH mutation is knocked in the mouse genome during embryogenesis, and it is possible that the absence of desacetyl-α-MSH and α-MSH during development contributes to the obese *Pomc*^tm1/tm1^ mouse phenotype. Furthermore, our model has global removal of desacetyl-α-MSH and α-MSH; therefore, we do not know whether the obese *Pomc*^tm1/tm1^ mouse phenotype is due to the removal of these peptides in the brain, in the periphery, or in both brain and periphery. POMC is most abundantly expressed in the pituitary gland and expressed in lower abundance in the arcuate nucleus of the hypothalamus, the brainstem, and in several peripheral tissues including skin, pancreas, intestine, heart, and reproductive organs [1]. However, our results indicate that pituitary and adrenal gland development and function are unaltered in our model, as supported by normal histology and corticosterone levels respectively. This does not reflect the EC_50_ for ACTH^QKQR^ that is 82-fold less than the EC_50_ for ACTH^KKRR^ coupling to mMC2R (Supplementary Figure S3). We hypothesize that the negative feedback regulation of pituitary pars distalis ACTH^QKQR^ production is significantly reduced resulting in a build-up of circulating ACTH^QKQR^. ACTH^QKQR^ is a full agonist (Supplementary Figure S3) at the mMC2R and this build-up of ACTH^QKQR^ would account for the normal corticosterone levels in the *Pomc*^tm1/tm1^ mouse. The development of a conditional *Pomc*^tm1/tm1^ mouse model should resolve these issues.

For over 15 years, we have understood that POMC-derived peptide hormones are required for regulation of food intake and energy expenditure but only now do we show that desacetyl-α-MSH and α-MSH are both key endogenous POMC-derived peptides responsible for mouse regulation of appetite, metabolism, and body weight. We hypothesize that physiological and environmental factors differentially regulate endogenous POMC-derived peptide processing leading to dynamic changes in abundance of each peptide produced in specific cell types in brain and pituitary, and these dynamic changes culminate in the regulation of appetite, metabolism and body weight. The recently discovered cannabinoid-induced ‘munchies’ mediated through POMC neurons in the brain, turning up the production of β-endorphin while turning down the production of α-MSH [46] supports this hypothesis. Our data could suggest that there is potential to exploit the naturally occurring POMC-derived peptides to treat obesity and type-2 diabetes, but this relies on first understanding the specific function(s) for desacetyl-α-MSH and α-MSH in the brain and the periphery.

## 5 Conclusion

We show here that desacetyl-α-MSH is indeed biologically active *in vivo* and like α-MSH it can reduce mouse body weight and fat mass. Therefore, our study highlights a need to understand how endogenous desacetyl-α-MSH and α-MSH levels correlate with measures of energy balance and whether there are distinct or redundant roles for these POMC-derived peptides *in vivo*.

## Authors contributions

K.G.M. was responsible for the overall experimental design in Auckland, New Zealand. A.C., S.L., and J.K.E. were responsible for the experimental design and data analysis for the metabolic cage experiments at The University of Texas Southwestern Medical Center, USA. S.B., K.H., K.V.B., A.S., and B.S. maintained the mouse colony at the University of Auckland, weighed mice, performed i.c.v. surgeries, euthanized mice, harvested tissues, analyzed data, and contributed to writing of the manuscript. A.M. trained and supervised researchers performing i.c.v. surgeries. K.H., C.B., and M.M. performed mass spectrometry on tissue and lysates and A.G. performed imaging mass spectrometry on pituitary. P.W.R.H., R.K., and M.A.B. synthesized native and mutant ACTH peptides. R.B. performed cell culture and adenylyl cyclase assays and analyzed this data. B.P. performed MRI and developed MRI data analysis. A.P.C., K.T., S.P., and K.H. performed testing of ACTH peptides *in vitro* and *in vivo*. K.G.M. with help from A.C., S.L., and J.K.E. wrote the manuscript that was reviewed by all authors.

## Conflict of interest

The authors declare that no conflicts of interest exist.

## Footnotes

11 The registered nomenclature for this mouse model.
