# Supplementary figures and images for: Desacetyl-α-melanocyte stimulating hormone and α-melanocyte stimulating hormone are required to regulate energy balance
Source: Mol Metab. 2017 Nov 24;9:207–16. doi: 10.1016/j.molmet.2017.11.008 (PMC5869732; doi:10.1016/j.molmet.2017.11.008)

Figure S1

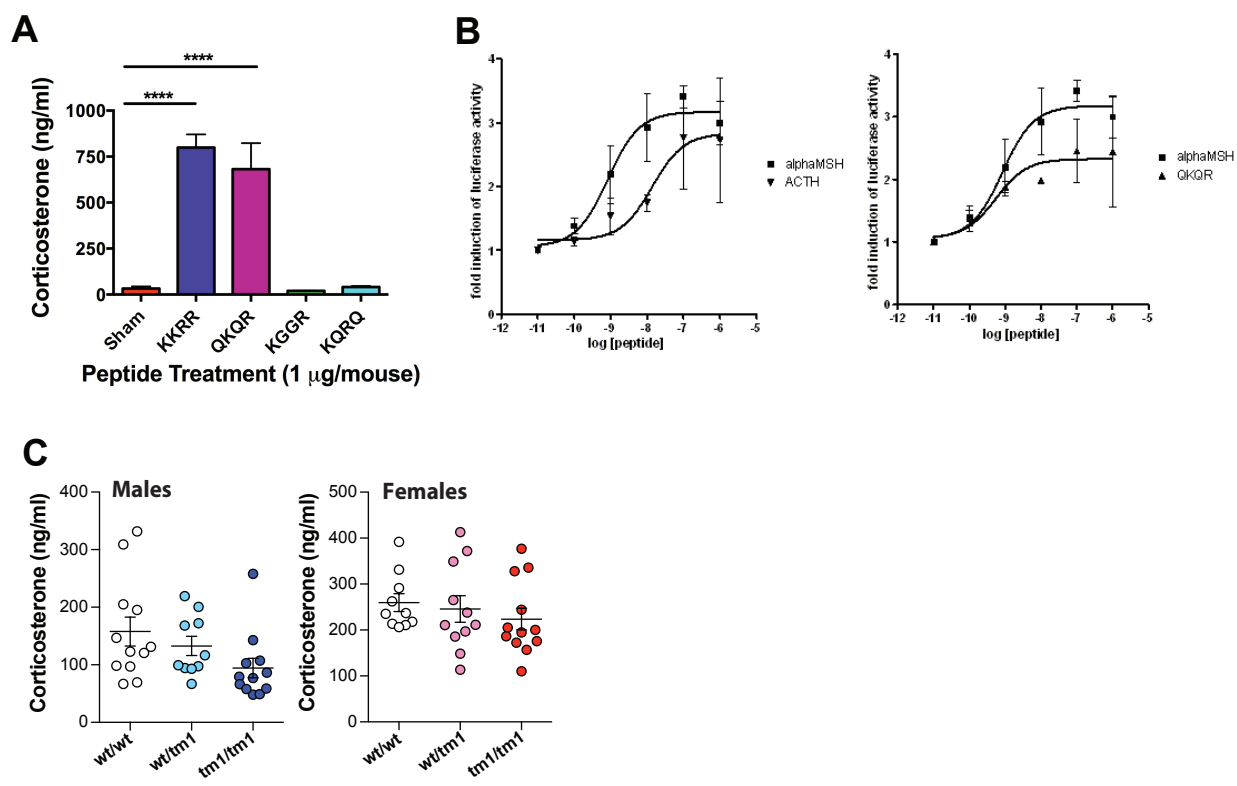

Supplement: Supplementary file 1 [file mmc1.pdf]

Figure S2

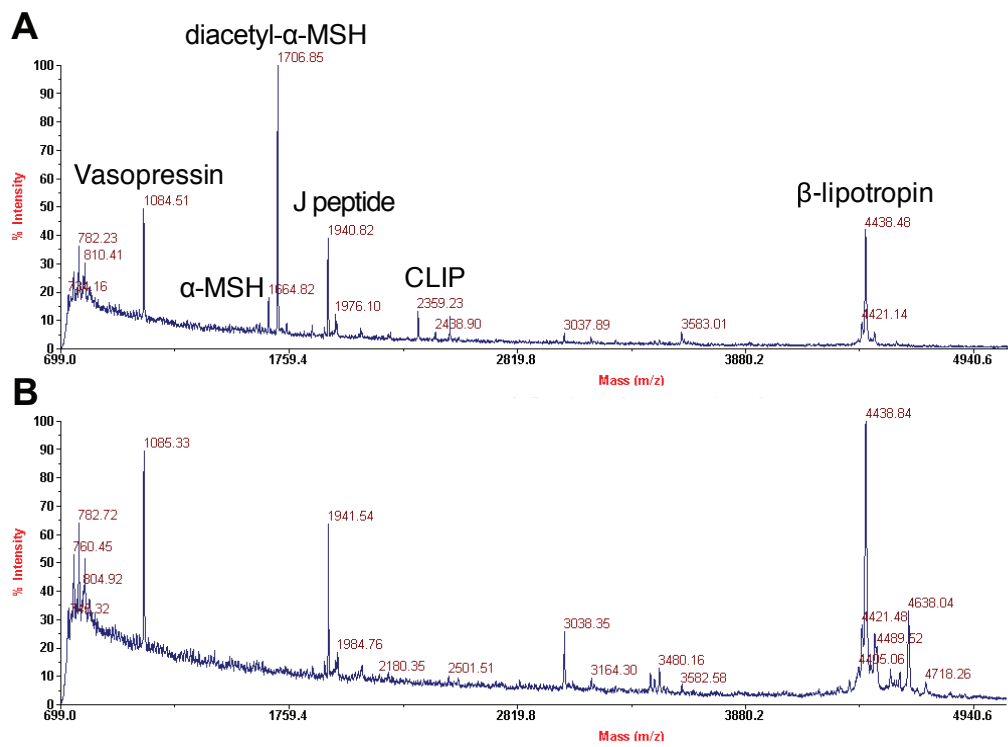

Supplement: Supplementary file 2 [file mmc2.pdf]

Figure S3

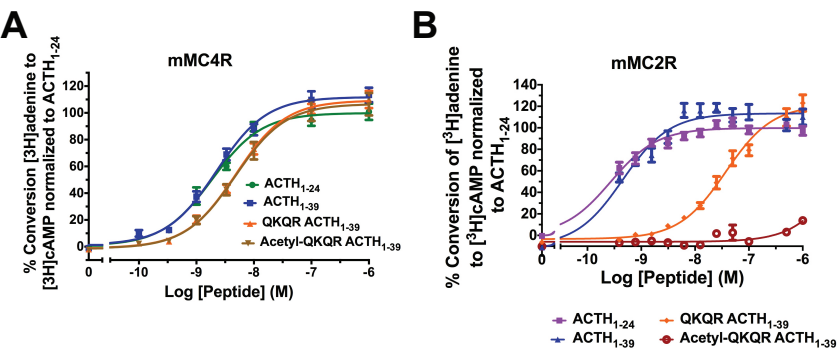

Supplement: Supplementary file 3 [file mmc3.pdf]

Figure S4

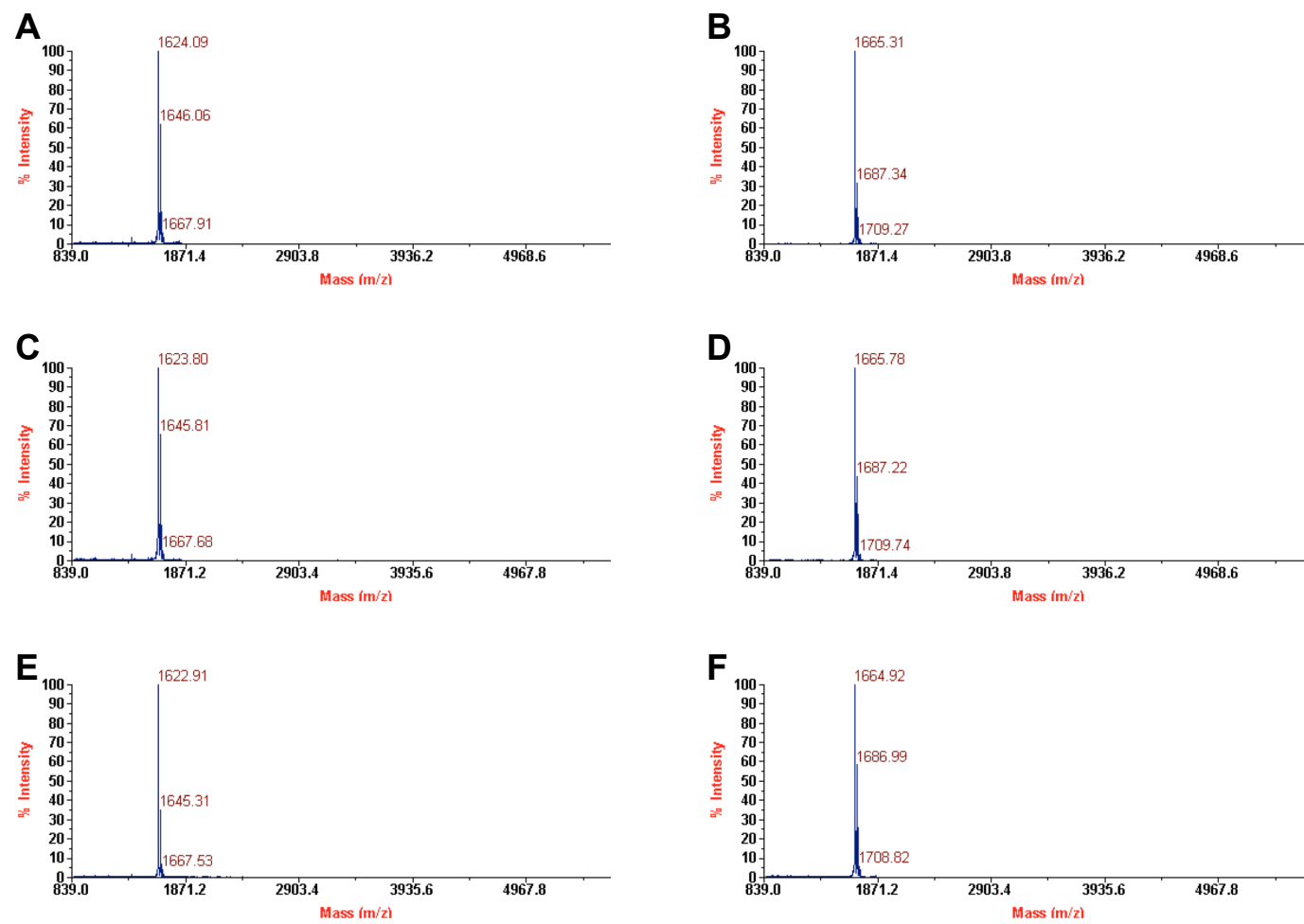

Supplement: Supplementary file 4 [file mmc4.pdf]
